# MicroRNA-152 regulates immune response via targeting B7-H1 in gastric carcinoma

**DOI:** 10.18632/oncotarget.15924

**Published:** 2017-03-06

**Authors:** Yaxin Wang, Di Wang, Gengchen Xie, Yuping Yin, Ende Zhao, Kaixiong Tao, Ruidong Li

**Affiliations:** ^1^ Department of Gastrointestinal Surgery, Union Hospital, Tongji Medical College, Huazhong University of Science and Technology, Wuhan 430022, China; ^2^ Department of Critical Care Medicine, Union Hospital, Tongji Medical College, Huazhong University of Science and Technology, Wuhan 430022, China

**Keywords:** gastric cancer, microRNA-152, B7-H1, immune response

## Abstract

MiR-152 has been reported may be involved in carcinogenesis in gastric cancer. However, its role has not been comprehensively investigated in gastric cancer. We found miR-152 in human gastric cancer tissues were significantly lower than that in matched adjacent normal tissues. Meanwhile, lower miR-152 was also found in gastric cancer cell lines. The stage, tumor size and lymph node metastasis rate were significant higher in low–miR-152 group in clinical patients. Furthermore, there was a marked correlation between the levels of miR-152 and B7-H1 mRNA in gastric cancer tissues. Mechanistically, miR-152 directly bind to B7-H1 3′ untranslated region in gastric cancer cell and inhibited B7-H1 expression. Functional study demonstrated that elevation of miR-152 enhanced T cells proliferation and effector cytokines production via inhibiting B7-H1/PD-1 pathway. In conclusion, our work identified a novel mechanism by which immune response is increased by expression of miR-152 via targeting B7-H1. MiR-152 may be a potential therapeutic approach for gastric cancer.

## INTRODUCTION

Gastric carcinoma (GC) is one of the most common malignancies in the world [1–3]. Despite improvements in diagnosis and treatments was developed in recent years, the advanced gastric cancer is still one of the most lethal cancers due to lack of effective therapies. To improve survival rate, recent studies have focused on immunotherapies by targeting B7 homolog 1 (B7-H1) or programmed death 1 (PD-1) in multiple cancers treatment [4, 5].

B7-H1, a type I transmembrane surface glycoprotein, is found on several types of immune cells surface such as dendritic cells and macrophages, but also on surface of multiple solid tumors [6–9]. B7-H1 is a member of superfamily of immunoglobulin B7 and act as the ligand of PD-1, which is important in immunomodulation [10–12]. The interaction of B7-H1 with PD-1 induces suppression of T cell's function and results in inhibition of immune response, which prevents tumor cells from being destructed by immune cells [13]. B7-H1/PD-1 mediated tumor cell immune escaping is important in cancer development and metastatic. Many studies have found that B7-H1 expression was associated with poor survival rate in cancer [14–17]. A promising treatment is blocking the immune checkpoint to augment endogenous antitumor immunity. Antibodies specified to PD-1/B7-H1 immune checkpoint were designed to restore endogenous anti-tumor response. Currently, it is reported that anti-PD-1 treatment profoundly improved the prognosis in certain lung cancer and metastatic melanoma [5]. Also, the efficacy of blocking PD-1 or B7-H1 in other cancer type, including gastrointestinal carcinoma, is in evaluation.

MicroRNAs are a type of endogenous small noncoding RNA ranging 20 to 25 nucleotides, which induces translational inhibition or degradation of targets by binding with specify sequence located at 3′-UTR (untranslated region) of mRNA [18]. Increasing studies have shown that microRNAs was involved in proliferation, apoptosis and immune response by targeting oncogenes and tumor suppressor genes [19, 20]. There were evidences that miR-152 was associated with gastrointestinal cancer. Song et al. found that miR-152 inhibited gastric cancer cells proliferation by targeting cholecystokinin-B receptor [21]. Zhai et al. reported miR-152 suppresses gastric cancer cell proliferation via targeting CD151 [22]. MiR-152 was reported down regulated in gastric cancer tissues compared to matched normal tissues and low expression of miR-152 was correlated with increased tumor sizes and stage [23]. Therefore, low expression of miR-152 is involved in carcinogenesis in gastric cancer. However, the underlying mechanism was still not fully investigated.

In our study, first we investigated expression of miR-152 in surgically resected human GC tissue and several GC cells lines. Then the association of expression of miR-152 and clinicopathologic characteristics were analyzed. Moreover, the association between expression of miR-152 and expression of GC immune check point was evaluated. We found expression of miR-152 was correlated with level of B7-H1. And next, the molecular mechanism which is involved in miR-152 regulating immune response was investigated.

## RESULTS

### Expression of miR-152 was decreased in gastric cancer tissues and cell lines compared to non-tumor control

**Figure 1:**
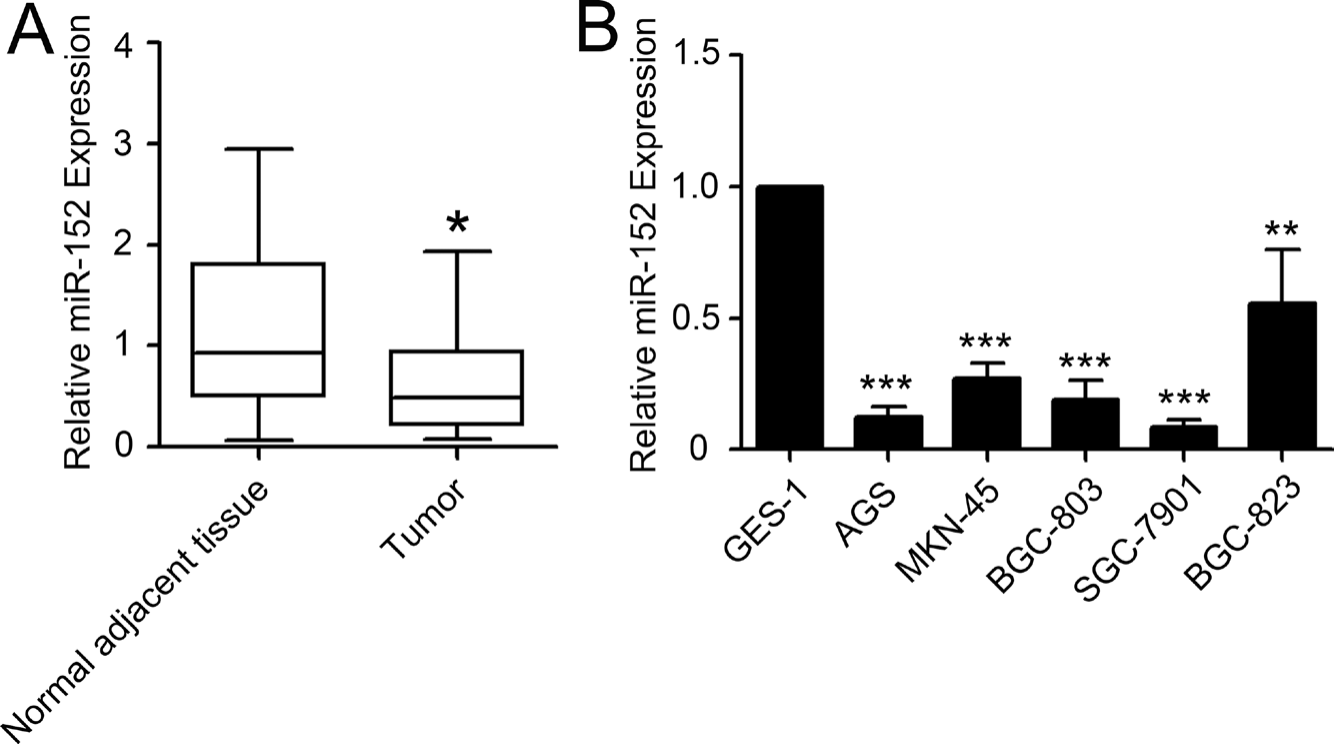
Expression of miR-152 in gastric cancer patients and cell lines Levels of miR-152 were measured by SYBR Green real-time PCR. (**A**) The levels of miR-152 in gastric cancer tissues was lower than matched normal adjacent tissues. (**B**) Several gastric cancer cell lines (AGS, MKN-45, BGC-823, SGC-7901 and BGC-803) also showed lower expression of miR-152 compared to normal gastric epithelial cell line (GES-1). Each samples were measured in triplicate and repeated three times. *P < 0.05; ^*^P < 0.01; ^**^*P < 0.001. Data were expressed as Mean ± SEM.

To explore miR-152 roles in gastric carcinoma, we used quantitative real-time polymerase chain reaction (qRT-PCR) to measure miR-152 level in cancer tissues and adjacent non tumor mucosa tissues. As shown in Figure 1A, miR-152 level significant decreased in gastric cancer tissues compared to normal control. In addition, we measured expression of miR-152 level in cell lines. As shown in Figure 1B, expression of miR-152 decreased markedly in five gastric cancer cell lines (AGS, MKN-45, BGC-803, GC-7901, and BGC-823) compared to normal gastric epithelium cell line (GES-1). Among them, SGC-7901 and AGS displayed the most reduction of miR-152. Thus they were chosen for further functional analyses on miR-152.

### Clinicopathologic features of miR-152 in gastric cancer patients

We divided all gastric cancer patients into two groups according to ratio of tumor/normal of miR-152. The high expression group was defined as cases with tumor/normal ratio over the median value and low expression group was defined as cases with tumor/normal ratio lower than median value. As shown in Table 1, we found that the low expression of miR-152 was associated with tumor size, stage and positive lymph node metastasis. However, we did not found any correlation between miR-152 and patient's age, gender, liver metastasis and tumor location.

**Table 1:** miR-152 expression and clinicopathologic factors

Factor	High expression (*n* = 21)	Low expression (*n* = 21)	*P* value
Age			*P* = 0.753
≥ 65	8	9	
< 65	13	12	
Gender			*P* = 0.190
Male	16	12	
Female	5	9	
Stage			*P* = 0.013
I + II	15	7	
III + IV	6	14	
Tumor size			*P* = 0.031
≥ 50 mm	7	15	
< 50 mm	14	6	
Liver metastasis			*P* = 0.549
absent	1	2	
present	20	19	
Lymph node metastasis			*P* = 0.030
absent	6	13	
present	15	8	
Tumor location			*P* = 0.469
Distal third	17	15	
Middle or proximal	4	6	

### Correlations among miR-152, B7-H3, B7-H1, B7-1 and B7-2 mRNA Levels in gastric cancer patients

**Figure 2:**
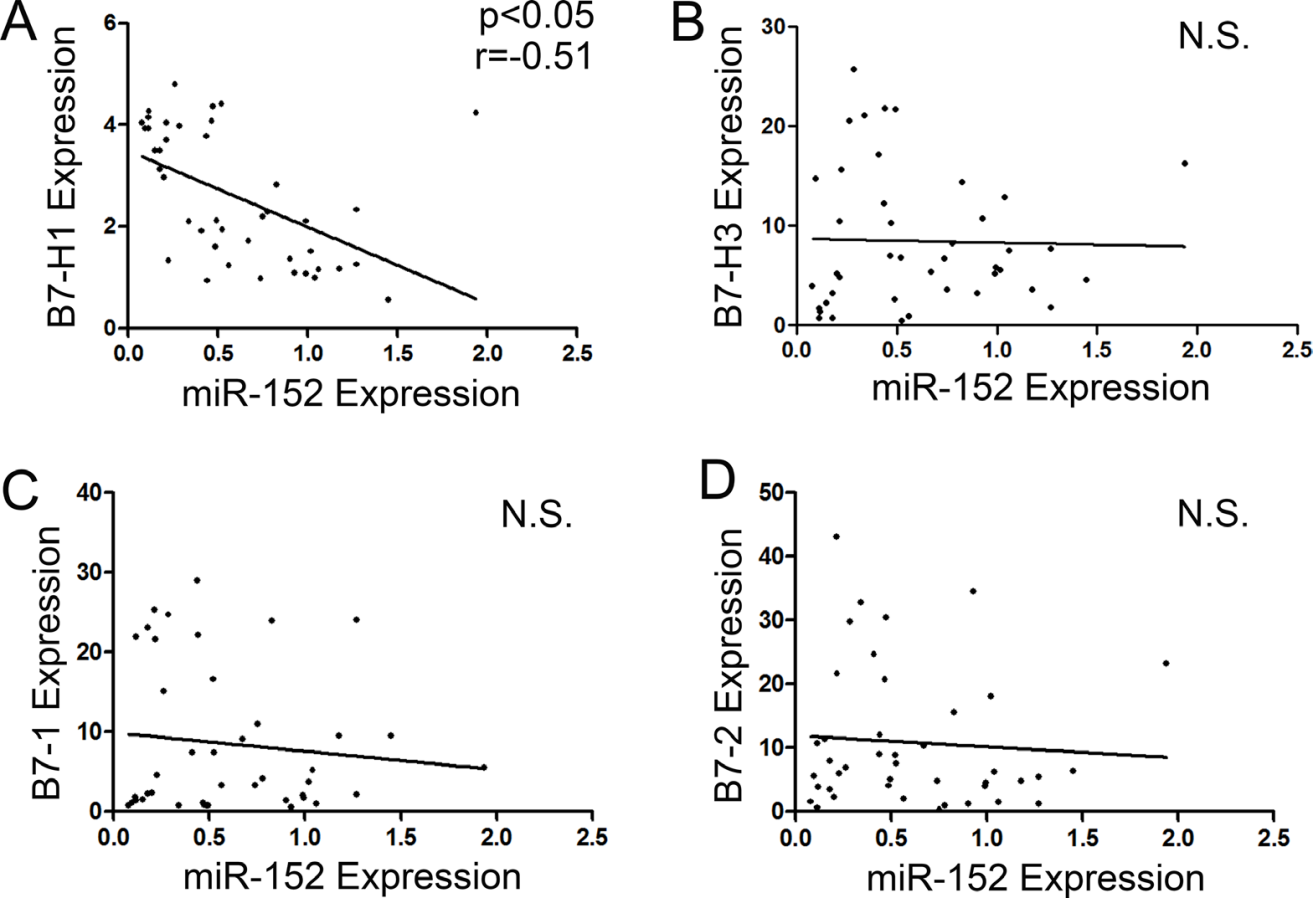
Correlation between miR-152 and mRNA expression levels of B7-1, B7-2, B7-H1 and B7-H3 (**A**) There was significant correlation between miR-152 and B7-H1 mRNA level (*p* < 0.05, *r* = −0.51). There was no significant correlation between miR-152 and mRNAs level of B7-H3 (**B**), B7-1 (**C**) and B7-2 (**D**). N.S. means no significance.

The expression levels of miR-152, B7-H3, B7-H1, B7-1 and B7-2 from 42 clinical gastric cancer samples were analyzed by qRT-PCR. As shown in Figure 2, there were no significant correlation between miR-152 and B7-H3, B7-1 and B7-2. However, there was marked correlation between miR-152 and B7-H1 (Figure 2A, *P <* 0.05, *r* = −0.51).

### B7-H1 expression was inhibited by transfection of miR-152 mimic in gastric cancer cell lines

**Figure 3:**
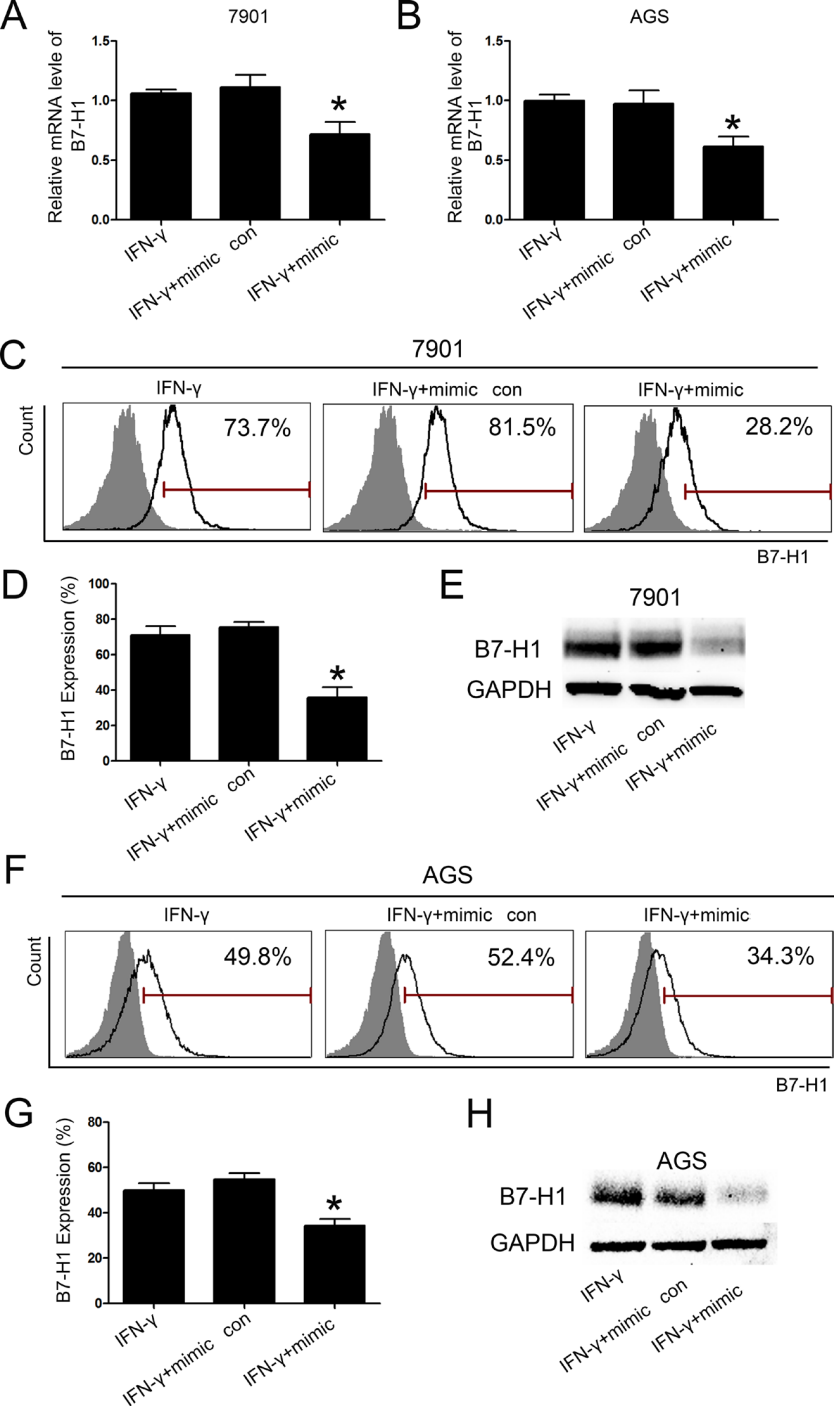
MiR-152 inhibited expression of B7-H1 in gastric cancer cell lines There was a significant reduction of B7-H1 mRNA expression with transfection of miR-152 mimic in SGC-7901 (**A**) and AGS (**B**). Flow Cytometry (**C** and **D**) and Western blot (**E**) demonstrated that there was a marked reduction of expression of B7-H1 with transfection of miR-152 mimic in SGC-7901. Also, expression B7-H1 was inhibited by transfection with miR-152 mimic in AGS cell line (**F**–**H**). All experiments were repeated three times. *P < 0.05. Data were expressed as Mean ± SEM. Mimic con means scramble mimic control group.

As expression of B7-H1 was negatively correlated with miR-152 levels in gastric cancer. Next, we tried to explore whether B7-H1 is potential target of miR-152. We used bioinformatics algorithm (www.targetscan.org) to screen target genes of miR-152 and found B7-H1 is putative target of miR-152. According to this rationale, B7-H1 was chosen for next analyses. As shown in Figure 3A and 3B, transfection of miR-152 mimic significantly reduced B7-H1 mRNA levels in SGC-7901 and AGS cell lines. Interferon-γ (IFN-γ) (20 ng/ml) was added 48h after transfection. Another 24 hours later, cells were collected and analyzed. Consistent with above result, the western blot and Flow Cytometry results showed that protein level of B7-H1 also markedly reduced after transfection with miR-152 mimic.

### Inhibition of miR-152 expression enhanced B7-H1 expression

**Figure 4:**
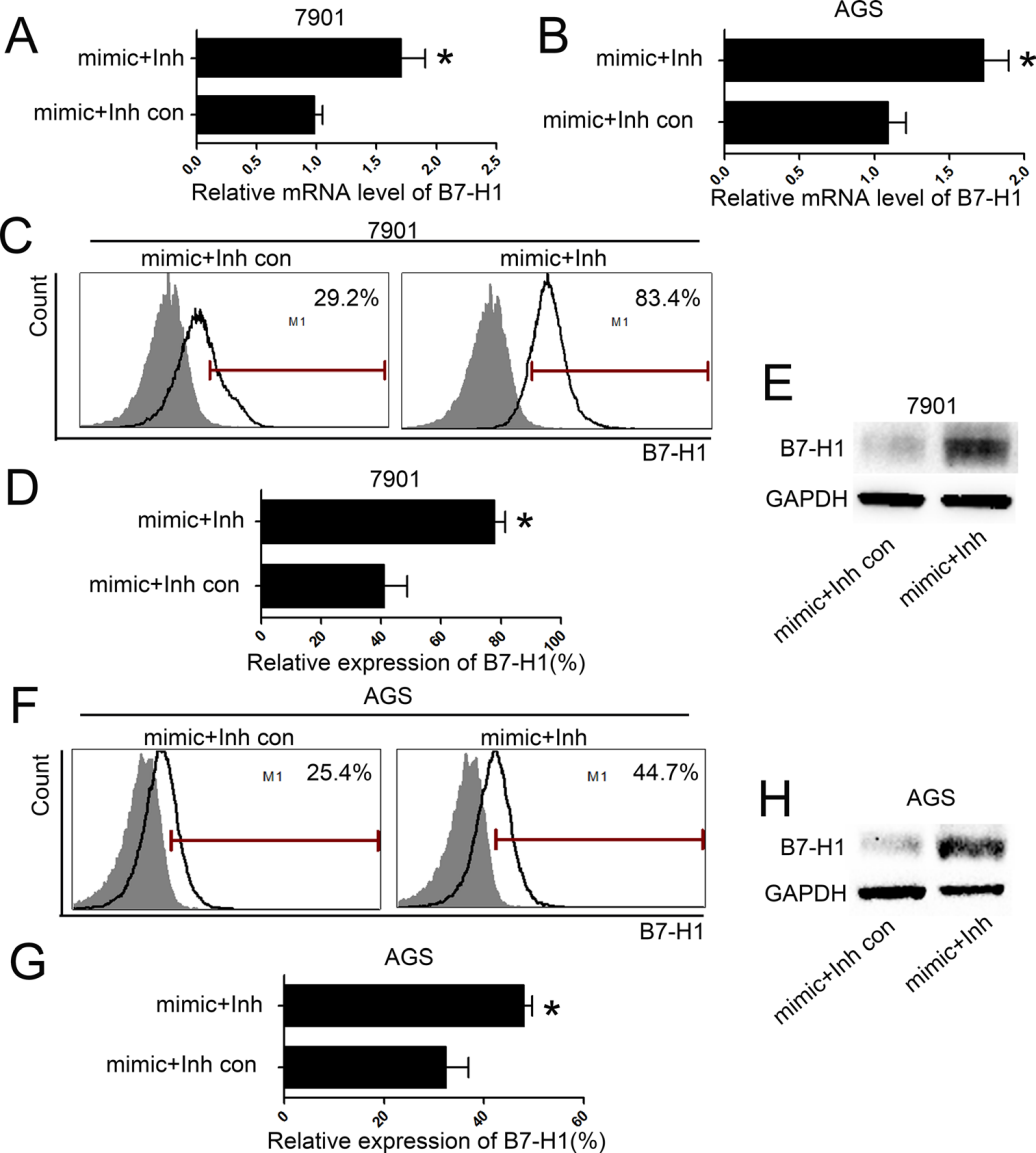
inhibition of miR-152 increased level of B7-H1 in gastric cancer cells MiR-152 inhibitor can significantly inhibited B7-H1 mRNA expression with co-transfection of miR-152 mimic in SGC-7901 (**A**) and AGS (**B**). Flow Cytometry (**C** and **D**) and Western blot (**E**) showed that miR-152 inhibitor increased expression of B7-H1 with co-transfection of miR-152 mimic in SGC-7901. Also, expression B7-H1 was increased by co-transfection with miR-152 mimic and inhibitor in AGS cell line (**F**–**H**). All experiments were repeated three times. *P < 0.05. Data were expressed as Mean ± SEM. Inh means miR-152 inhibitor; Inh con means miR-152 inhibitor control.

MiRNA inhibitor is chemically modified inhibitor to target and inhibit expression of specified miRNA. Here we used miR-152 mimic and inhibitor to co-transfect AGS and SGC-7901 cells. IFN-γ (20 ng/ml) was added and cells were collected as above. As shown in Figure 4, co-transfection of specified inhibitor and miR-152 mimic can significantly increase B7-H1 mRNA expression. Also, the protein level of B7-H1 markedly increased when cells were co-transfected with mimic and inhibitor. However, the control inhibitor group showed no such effects.

### B7-H1 is a direct target of miR152

**Figure 5:**
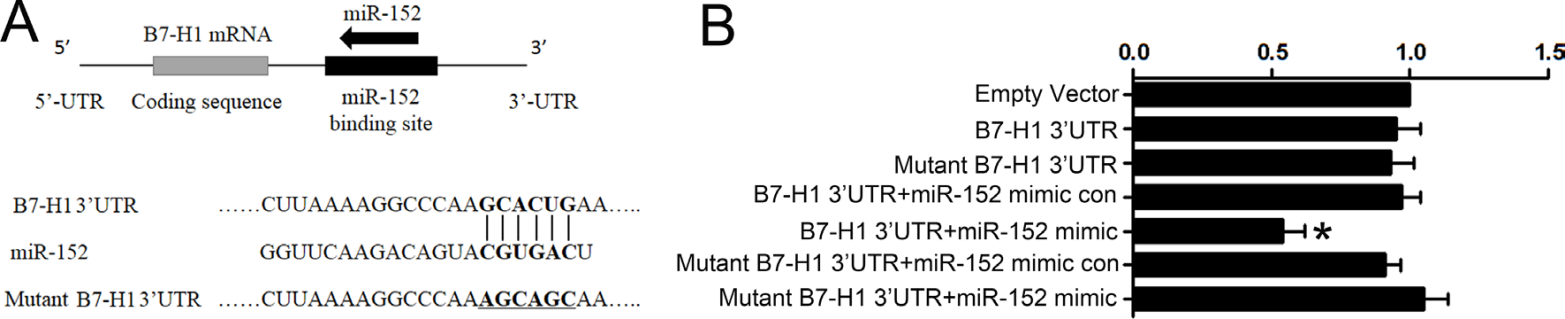
MiR-152 reduced luciferase activity via directly binding to 3′-UTR of B7-H1 Binding site and sequence of the B7-H1 within luciferase report vector (**A**). Relative luciferase activity in cells co-transfected with miR-152 or scramble control and a luciferase vector encoding luciferase gene fused wild type (WT) or mutant B7-H1 3′-UTR (**B**). The WT B7-H1 3′-UTR+miR-152 group showed significant reduction of luciferase activity (B). *P < 0.05 vs. Empty vector group. All experiments were repeated three times. Data were expressed as Mean ± SEM. Mimic con means scramble mimic control; 3′UTR means 3′ untranslated region.

To confirm whether B7-H1 is a direct target of miR-152 in GC cells, the luciferase report assay was performed. We co-transfected SGC-7901 with miR-152 and a reporter vector encoding luciferase which is fused with 3′-UTR of B7-H1 gene (Luc-B7-H1 vector). As shown in Figure 5, luciferase activity was significantly inhibited when cells were transfected with miR-152 mimic and Luc-B7-H1 vector. However, co-transfection with miR-152 mimic scramble control and Luc-B7-H1 vector or empty vector showed no significant effect to luciferase activity. Moreover, mutation of predicted binding site of 3′-UTR of B7-H1 marked rescued activity of luciferase. Therefore, we confirmed that miR-152 could bind directly to B7-H1 3′-UTR region and inhibit B7-H1 expression.

### MiR-152 increased T cells proliferation and function via inhibition of B7-H1

**Figure 6:**
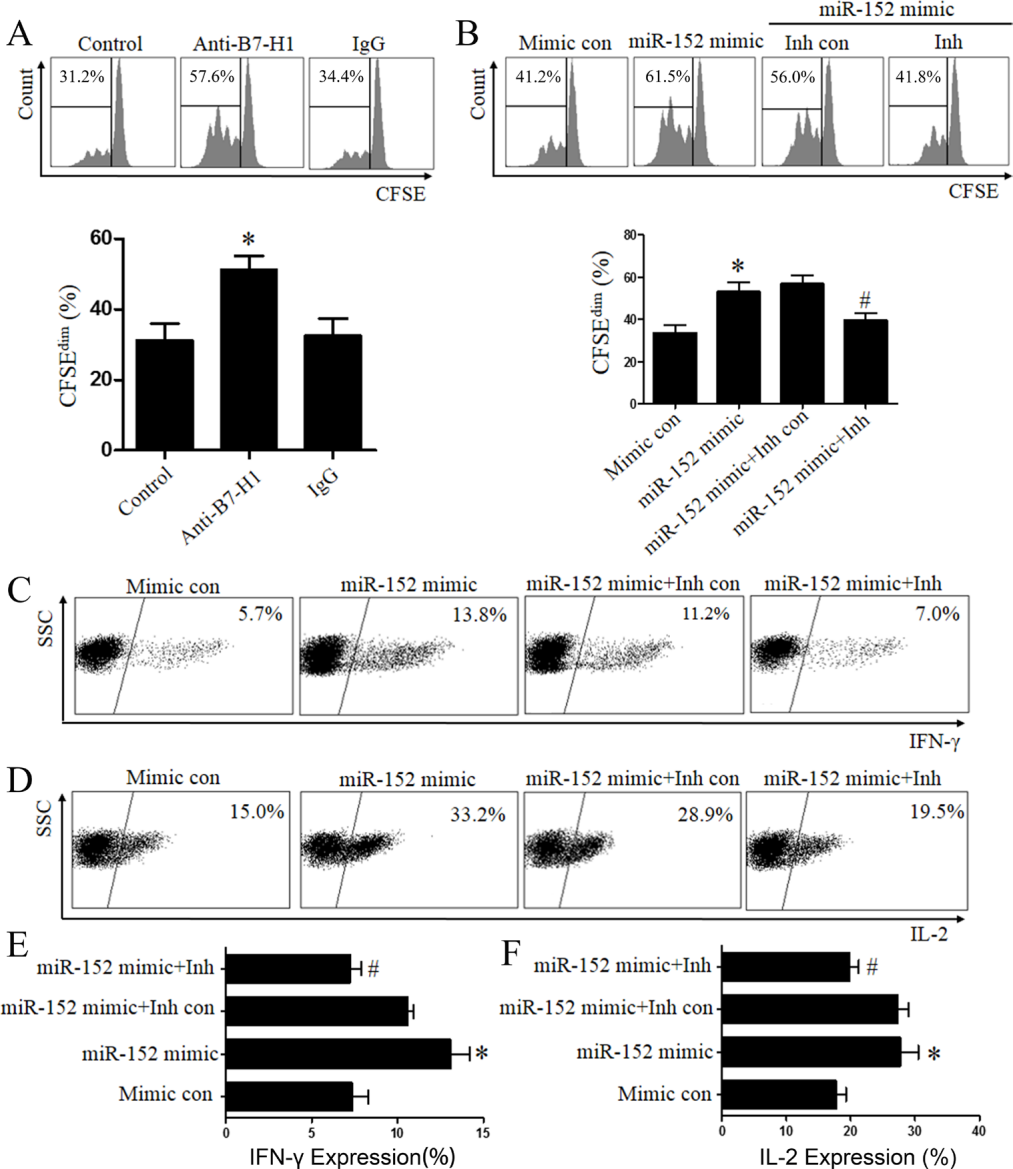
MiR-152 restored T cells function via inhibition of B7-H1 Neutralizing anti-human B7-H1 antibody restored proliferation of T cells while isotype mAbs showed no such effect (**A**). Transfection of miR-152 mimic restored T cells proliferation (**B**) and co-transfection specified miR-152 inhibitor with mimic can eliminate such effect (B). Cytokines (IFN-γ and IL-2) production increased after co-cultured with gastric cancer cells (SGC-7901) transfected of miR-152 mimic and decreased after co-cultured with cells co-transfected with specified miR-152 inhibitor with mimic (**C**–**F**). ^#^*P <* 0.05 versus the miR-152 mimic+Inh con group. *P < 0.05 versus the mimic con group or control group. All experiments were repeated three times. Data were expressed as Mean ± SEM. Mimic con means scramble mimic control. Inh con means miR-152 inhibitor control; Carboxyfluorescein succinimidylester (CFSE).

It is widely accepted that B7-H1 is able to induce T cells anergy. The T cells co-culture assay was applied to confirm whether miR-152 increased immune response. As shown in Figure 6A, T cells proliferation was inhibited by expression of B7-H1. However, blockade of B7-H1 by specified B7-H1 antibody (10 μg/ml, Biolegend) was able to increase proliferation of T cells while isotype IgG showed no such effect, which indicated that T cells proliferation was inhibited by overexpression of B7-H1. Here we detected whether miR-152 was able to increase T cells function by inhibiting B7-H1. As shown in Figure 6B, transfection of mimic of miR-152 significantly increased proliferation ability of T cells while scramble mimic control had no such effect. And co-transfection of miR-152 mimic with inhibitor showed inhibition of T cells proliferation compared to transfection of miR-152 mimic with scramble inhibitor group. Also, the production of cytokines, which represented function of T cells, was measured. Similarly, transfection of mimic of miR-152 significantly augmented interleukin-2 (IL-2) and IFN-γ production and co-transfection of miR-152 mimic with inhibitor inhibited rate of IL-2 and IFN-γ positive T cells (Figure 6C–6F). All those indicated that miR-152 increased immune response by inhibiting B7-H1.

## DISCUSSION

Previous studies have demonstrated that miR-152 acts as a tumor suppressor [21, 22, 24, 25]. However, the involvement of miR-152 in tumor immune is still poorly understood. Our finding from present study defined a new role for miR-152 and suggested that miR-152 specifically activates immune response via targeting B7-H1. In the present study, lower miR-152 level was significantly related to clinicopatholgic parameters such as increasing tumor size, advanced pT stage and higher lymph node metastasis rate (Table 1). It is widely accepted that tumor size, pT stage and lymph node metastasis rate are pivotal prognostic factors in gastric cancer patients. We found that there was a significant inverse correlation between miR-152 and B7-H1 expression in gastric cancer samples (Figure 2). Hence we speculated that miR-152 might be associated with expression of B7-H1 in gastric cancer.

MiRNA-152 mimic and inhibitor were used to explore the specific interaction between miR-152 and B7-H1. Here we found that transfection of miR-152 mimic reduced B7-H1 expression in SGC-7901 and AGS gastric cell lines (Figure 3). These finding suggested that miR-152 inhibited B7-H1 expression. Moreover, we co-transfected gastric cancer cell lines with specified miR-152 inhibitor and mimic and found that could restore expression of B7-H1 in gastric cancer cell lines (Figure 4), which indicated miR-152 can specifically inhibit expression of B7-H1. Furthermore, dual-luciferase reporter assay demonstrated that miR-152 can directly bind with 3′-UTR of B7-H1. Collectively, all these data indicated that expression of B7-H1 is inhibited by miR-152 and thus loss miR-152 may result in development of cancer

T cells is important in immunologic surveillance and loss of function of T cells may lead to tumor immune escaping [26]. Many studies suggested that activation of B7-H1/PD-1 signaling induced T cells exhaustion, a process broadly described as dysfunction and deletion of specified T cells. T cells exhaustion is accompanied with decreasing production of several cytokines such as IFN-γ and IL-2 [27]. Consequently, blockade B7-H1/PD-1 pathway restored function of T cells in several types of tumor and showed anti-tumor response by modulating tumor microenvironment [4, 13]. In present study, transfection of miR-152 can significantly restore proliferation of T cells, suggesting that miR-152 have a direct effect on immune response by decreasing expression of B7-H1, which can be explored therapeutically (Figure 6). In accordance with the recovery of proliferation, the production of cytokines including IFN-γ and interleukin-2 were increased by transfection of miR-152 mimic (Figure 6). From our results, we revealed new function of miR-152 and displayed that miR-152 can reactivate T cells response *in vitro*. Therefore, the miR-152 may have therapeutic potential to increase immune response, as well as to inhibit growth of tumor in gastric cancer.

It is likely that further research into microRNA-152 might shed the light on the development of drug and therapeutic strategies for treatment of gastric cancer. However, a limitation for this study is the fact that an investigation of correlation between miR-152 expression and patients’ survival is needed. And the influence to immune response *in vivo* should be further investigated.

In conclusion, our study provided data that miR-152 was significantly decreased in gastric cancer tissues and cell lines. Lower miR-152 is associated with poor stage, increasing tumor size and higher positive lymph node metastasis. MiR-152 could bind with 3′-UTR of B7-H1 and inhibited its expression. Subsequently, enforced miR-152 expression enhanced T cells proliferation and function via targeting immune checkpoint molecular B7-H1. Our results therefore suggests miR-152 is a potential prognostic and therapeutic marker for gastric cancer and identify a novel mechanism by which tumor immune response is regulated by miR-152 via B7-H1.

## MATERIALS AND METHODS

### Tissue samples

All human gastric cancer tissue and corresponding adjacent normal tissue were obtained from 42 patients who underwent a surgical resection without preoperative treatment at Union hospital of Huazhong University of Science and Technology, after receiving adequate informed consent. Fresh samples were preserved by snap-frozen in liquid nitrogen immediately after resection and then stored in −80°C. The present study was approved by the Research Ethics Committee of Huazhong University of Science and Technology.

### Cell line and culture condition

Human gastric cancer cells lines (AGS, SGC-7901, MKN-45, BGC-823, and BGC-803) and human immortalized gastric epithelial cell line (GES-1) were obtained from Cell Bank, Chinese Academy of Science (Shanghai, China). SGC-7901, BGC-803 and BGC-823 were cultured in RPMI-1640 culture medium (Invitrogen, USA). AGS was cultured in F-12K medium (Invitrogen, USA). MKN-45 and GES-1 were maintained in Dulbecco modified Eagle medium (Invitrogen, USA). All medium contained 10% fetal bovine serum (FBS). All cells were grown at 37°C with 5% CO_2_ in a humidified incubator.

### RNA isolation and polymerase chain reaction

The total RNA extraction was carried out using TRIzol reagent (Takara, Japan) according to manufacturer's instruction. The reverse transcription was performed using Primescript Reverse Transcription Regent Kit (Takare, Japan) according to protocol of manufacturer. Quantitative real-time polymerase chain reaction (qRT-PCR) was performed using SYBR Green PCR Master Mix Kit (Takara, Japan) according to manufacturer's instruction. A 7500 HT analyzer (Applied Biosystems, Foster City, CA, USA) was used in qRT-PCR process. The expression of microRNA-152 and mRNAs level were determined based on CT value and normalized to U6 or GAPDH levels, respectively. The miR-152 and U6 primers were obtained from Ribobio (Guangzhou, China). The mRNA primers are listed: B7-1 Forward: 5′-GCAGGG AACATCACCATCCA-3′, Reverse: 5′-AGGTGAGGCTC TGGAAAACC-3′; B7-2, Forward: 5′- GGGCCGCACA AGTTTTGATT −3′, Reverse: 5′- AGGCCGCTTCTTCTT CTTCC −3′. B7-H3 Forward: 5′- CTTCGTGAGCATC CGGGATT −3′, Reverse: 5′- GTGAAACTGTGCACCAGC TG-3′; B7-H1 Forward: 5′- CAAGGCCGAAGTCATCTG GA −3′, Reverse: 5′-CCATTTTCAGTGCTTGGGCC-3′; GAPDH Forward: 5′- CGTGGAAGGACTCATGAC CA −3′, Reverse: 5′- GGTCTTACTCCTTGGAGGCC −3′. All qRT-PCR reactions were performed in triplicate. The relative amount of miR-152, B7-1, B7-2, B7-H1 and B7-H3 were calculated using the 2^–ΔΔCt^ method described before [28].

### Functional assay

Before co-culture, SGC-7901 was transfected with miR-152 mimic, inhibitor or mimic and inhibitor for 48 hours according to manufacturer's protocol then IFN-γ (20 ng/ml) was added for another 24 hours. After that, cells was treated with Mitomycin C (10 μg/ml, Sigma) for 1.5 hours. Meanwhile, T cells were cultured alone in the presence of anti-human CD3 (2.5 μg/ml, BD Biosciences) and anti-human CD28 (2.5 μg/ml, BD Biosciences) for 2 days then T cells (5*10^5^) were co-cultured with above SGC-7901 (5:1) in the presence of anti-human CD3 (2.5 μg/ml, BD Biosciences) and anti-human CD28 (2.5 μg/ml, BD Biosciences) for 2 days. Then T cells were collected and stained with flurochrome-conjugated-specific antibodies extracellularly. Then T cells were fixed and permeabilized by using Perm/Fix solution (eBioscience, USA) according to the manufacturer's instruction. After that, the intracellular cytokines were stained by flurochrome-conjugated-specified anti-human IL-2 and IFN-γ antibody (eBioscience, USA) according to the manufacturer's instruction.

### T-cell proliferation assay

CD4 positive T cells were obtained from peripheral mononuclear using paramagnetic beads (StemCell Technology, Canada) according to manufacturer's protocol. Cells purity was > 90% confirmed by low Cytometry (LSR II, Becton Dickinson). To determine cell division, CD4^+^ T cells were labeled using Carboxyfluorescein succinimidylester (CFSE, Biolegend, USA) according to manufacturer's instruction. After labeled, T cells were cultured alone in the presence of anti-human CD3 (2.5 μg/ml BD Biosciences) and anti-human CD28 (2.5 μg/ml, BD Biosciences) for 2 days. Before co-culture, SGC-7901 was treated as described in upper functional assay section. Then CD4 positive T cells were collected and incubated with treated SGC-7901 (5:1) for another two days in the presence of anti-human CD3 (2.5 μg/ml, BD Biosciences) and anti-human CD28 (2.5 μg/ml, BD Biosciences). Cell division of CD4^+^ T cells was analyzed by Flow Cytometry (LSR II, Becton Dickinson).

### B7-H1 expression by flow cytometry

Surface B7-H1 expression was measured by Flow Cytometry using allophycocyanin (APC)-conjugated anti-human B7-H1 (clone: MIH1) antibody (BD bioscience, USA). After washing with Phosphate Buffered Saline (PBS) containing 2% fetal bovine serum (FBS), cells were incubated with B7-H1 antibody or matching isotype control antibody for 40 minutes. Then washed with PBS containing 2% FBS twice and fixed with 2% paraformaldehyde in PBS. B7-H1 expression was analyzed using Flow Cytometry (LSR II, Becton Dickinson).

### Luciferase report assay

SGC-7901 cells were grown in 96-well plates at 3 × 10^4^ cells per well. 24 hours later, cells were cotransfected with miR-152 mimic or mimic control (scrambled control miRNA) with empty control vector or expression vectors containing luciferase gene fused to the wild type (WT) or mutant miR-152 binding site B7-H1 3′UTR (RiboBio, Guangzhou, China) using HiPerFect Transfection Reagent (Qiagen, Hilden, Germany). 48 hours after transfection, firefly and Renilla luciferase activities were measured by a Dual-luciferaseReporter Assay System (Promega, Madison, WI, USA) according to the manufacturer's instruction.

### Western blot analysis

Cells were collected and lysed using Radio Immunoprecipitation Assay (Beyotime Biotechnology Company, China) with proteinase inhibitor according to manufacturer's protocol. Briefly, the lysate was centrifuged at 12000 g for 15min at 4°C. Then supernatant was collected and protein concentration was measured using BCA Protein Assay Kit (Beyotime Biotechnology Company, China). 20 ug protein of each sample was separated in 10% polyacrylamide sodium dodecyl sulfate gels. Then protein was transferred to to polyvinylidene fluoride membrane and blocked with 5% fat-free milk for 3 hours with Tris-buffered saline Tween-20 (TBST) (Beyotime, Beijing, China) at room temperature. The membranes were incubated with antibodies against B7-H1 (1:1000, Cell Signaling Technology, USA), GAPDH (1:3000, 6C5, Abcam, USA) overnight at 4°C and washed 3 times (10 minutes per time) after incubation. After that, The membranes were processed in secondary antibodies conjugated with horseradish peroxidase (HRP) for 1–2 hours at dilution of 1:4000. After washing as previously, the protein was detected using an ECL Kit (Beyotime Biotechnology Company, China) and images were captured by the UVP imaging system. B7-H1 expression was normalized to endogenous reference GAPDH.

### Statistical analysis

All data were showed as means ± standard error of the mean (SEM). The relationship between the expression of miR-152 and clinicopathologic features was analyzed by χ2 tests. Pearson's coefficient correlation was applied for analyzing associations between levels of miR-152 and mRNA levels of B7-H1, B7-1, B7-2 and B7-H3. The miR-152 expression level in cancer tissues and its mormal control were analyzed by Student's *t-test*. Statistical analysis was performed by one-way analysis of variance (ANOVA) plus Student-Newman-Keuls post hoc analysis. Statistical analyses were done using the SPSS software version 17.0 (SPSS Inc, Chicago, IL). *P value* less than 0.05 was considered statistically significance.
